# An Imidazo[2,1‐b][1,3,4]thiadiazole Derivative Inhibits the Virulence Factor α‐Hemolysin by Blocking the Pullout of Its Stem Domain

**DOI:** 10.1002/cmdc.202501098

**Published:** 2026-02-23

**Authors:** Vadim S. Korotkov, Peer Lukat, Raffaella Di Lucrezia, Aditya Shekhar, Carsten Degenhart, Randi Diestel, Ursula Bilitewski, Klaus Dinkel, Wulf Blankenfeldt, Mark Brönstrup

**Affiliations:** ^1^ Chemical Biology Helmholtz Centre for Infection Research Braunschweig Germany; ^2^ Structure and Function of Proteins Helmholtz Centre for Infection Research Braunschweig Germany; ^3^ Lead Discovery Center Dortmund Germany; ^4^ Compound Profiling and Screening Helmholtz Centre for Infection Research Braunschweig Germany; ^5^ German Center for Infection Research (DZIF) Site Hannover‐Braunschweig Germany; ^6^ Center of Biomolecular Drug Research (BMWZ) Leibniz Universität Hannover Germany

**Keywords:** antivirulence, antibacterials, high‐throughput screening, structural biology, structure–activity relationships

## Abstract

*Staphylococcus aureus* is a major human pathogen responsible for severe infections that necessitate alternative therapeutic strategies. Its key virulence factor α‐hemolysin (Hla) mediates host cell damage via pore formation, making it an attractive target for antivirulence interventions. Here, we report the development of a high‐throughput cellular assay measuring toxin‐induced calcium influx. Its application led to the identification of thiadiazole‐based small molecule inhibitors of Hla. Structure–activity relationship studies with 18 analogs led to inhibitors with a cellular potency up to 5.4 µM. X‐ray crystallography of Hla in complex with compound **1** revealed that the thiadiazole bound a hydrophobic pocket at the interface of the amino latch and prestem domains, exerting a dual mechanism that blocks stem loop unfolding as well as membrane attachment. These findings introduce thiadiazoles as a novel chemical class of antivirulence therapeutics against *S. aureus* infections.

## Graphical abstract

A high‐throughput cellular screen based on Ca^2+^ influx in U937 monocytic cells identified thiadiazoles as small‐molecule inhibitors of *α*‐hemolysin, a key virulence factor of *Staphylococcus aureus*. The thiadiazole **1** prevents pore formation by a dual mechanism that prevents stem loop unfolding as well as membrane attachment.

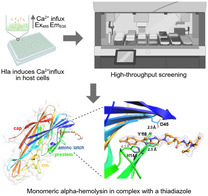

## 1 Introduction

Bacterial infections represent a serious, global health issue that is exacerbated by the rise of antibiotic‐resistant strains. Among the most harmful human pathogens is *Staphylococcus aureus*, which causes severe infections in several human body sites, including the skin, soft tissues, bone, lung, bloodstream, and heart as well as in implants [1].

Invasive infections of *S. aureus* come with high morbidity and mortality irrespective of effective antibiosis [2]. The fact that the absence of resistance against antibiotics is not sufficient to assure satisfactory clinical outcome implies that new treatment concepts that go beyond antibiotics are required. In this context, it has been hypothesized that the inhibition of host‐directed virulence factors secreted by the pathogen can complement or, under certain conditions, even replace the use of antibiotics. Advantages of virulence blockers are that they preserve the commensal microbiota and do not exert a selection pressure that fosters the formation of resistance. Such nontraditional approaches have therefore attracted considerable attention, and first antibody‐based toxin neutralizers have gained market approval [3, 4, 5].

The protein *α*‐hemolysin (aka *α*‐toxin or Hla) is the dominant bacterial virulence factor in *S. aureus* pneumonia [6, 7, 8]. Hla is secreted by the bacteria as a monomer that then assembles in host cell membranes to form a heptameric ionophoric pore. Ion leakage through such pores is cytotoxic. A substantial amount of preclinical and epidemiological data on the driving role of Hla in pneumonia has been published: Hla knock‐out strains were found to be largely avirulent to mice, ferrets, and rabbits in establishing pneumonia [9], and elevated natural anti‐Hla antibody levels in human cohorts were found protective from disease [10, 11]. Moreover, two monoclonal antibodies directed against Hla showed efficacy in subgroups in clinical proof‐of‐concept studies [12, 13]. Thus, Hla represents a highly attractive target that is validated by human data. Because small molecules are expected to exert superior pharmacokinetic properties with respect to building rapid exposure in the lung, we aimed to find small molecule drugs targeting Hla. We recently found that quinoxalinediones (QDS) inhibited Hla with high potency in cellular models [14]. By improving survival and bacterial load in lung infection models, the QDS demonstrated that the inhibition of large toxins by small molecules is feasible. Besides, flavones [15, 16, 17] or cyclodextrins [18, 19, 20] have been shown to interact with Hla or modulate its action. In this study, we report thiadiazoles as a structurally distinct class of Hla inhibitors. We present a high‐throughput assay that led to their discovery, initial structure–activity relationships, their ADME properties, and an X‐ray co‐crystal structure that shows their binding mode to Hla at atomic resolution.

## 2 Results and Discussion

### 2.1 Development of a High‐Throughput Screening Assay for Hemolysin Inhibitors

**FIGURE 1:**
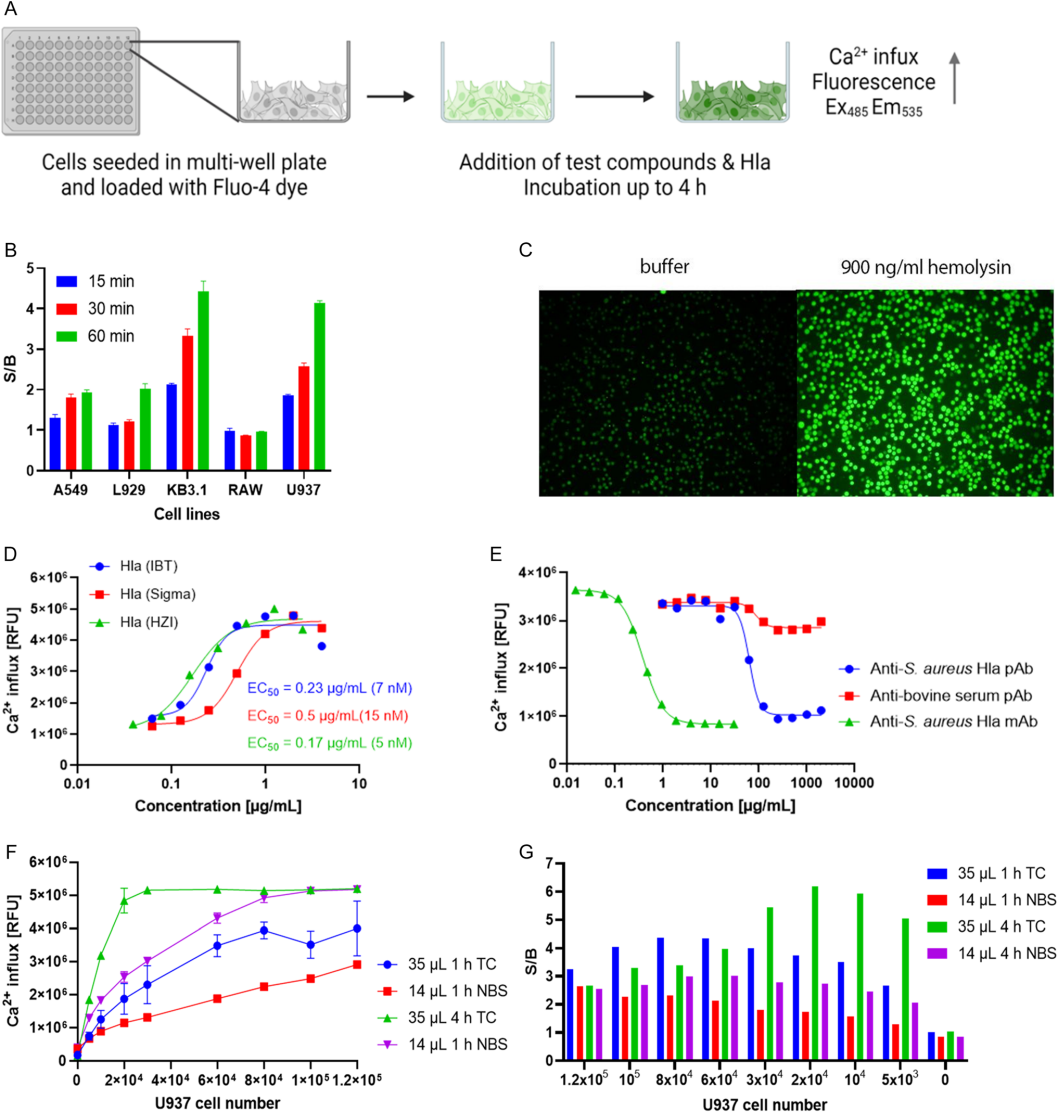
Development of the Ca^2+^ influx assay**.** (A) Diagrammatic representation of assay workflow. Created with BioRender.com. (B) Response of various cell types toward 3.3 µM Hla‐induced Ca^2+^ influx shown as signal height and signal background (S/B) ratio with different cell lines. (C) Fluorescence microscopy of cells incubated with 900 ng/mL (27 nM) Hla (right), or with buffer control (left). (D) Dose‐dependent increase of Ca^2+^ influx by Hla proteins from three sources. (E) Reversal of Hla‐induced Ca^2+^ influx by polyclonal (blue) and monoclonal (green) anti‐Hla antibodies. An unspecific anti‐bovine serum antibody (red) did not reverse influx. (F,G) Optimization of cell number per well and incubation time in two different 384 well plates with respect to signal height and signal background (S/B) ratio with 30 nM Hla: 20,000 cells/well in TC plates resulted in sufficiently high signals and good S/B, when cells were incubated for 4 h at room temperature. TC: Tissue culture treated 384 well plates with working volume of 5–40 µL; NBS: nonbinding surface 384 well plates with working volume of 2–20 µL. Final assay volumes were 35 and 14 µL, respectively.

In order to discover small molecule inhibitors of hemolysin, we developed a cellular assay with a phenotypic readout that captured Hla‐dependent effects (Figure 1). The assay is based on Hla's ability to induce an influx of Ca^2+^ ions in mammalian cells. By visualizing intracellular Ca^2+^ levels with the Fluo‐4 dye, active compounds were detected by the inhibition of Hla‐induced influx (Figure 1A). Because Hla is active in a large variety of cell types, we first selected an appropriate immortalized cell line for screening among human lung epithelial cells (A549), mouse fibroblasts (L929), a subclone from human cervical HeLa cells (KB3.1), mouse macrophages (RAW 264.7), and human monocytes (U937). In order to assure a high throughput of the assay, short exposure times of 15, 30, or 60 min were chosen. Under these conditions, 3.3 µM Hla led to signal‐to‐background (S/B) ratios of 1.93, 2.02, and 0.96 after 60 min in A549, L929, and RAW cells, respectively (Figure 1B). Higher S/B ratios of 4.43 and 4.15 were found for KB3.1 and for U937 cells, respectively. The latter were preferred, because they are in suspension and can be readily adapted to a high‐throughput screening format. Moreover, their pro‐monocytic immune cell characteristic makes U937 cells a physiologically relevant model for investigating Hla‐dependent cellular damage.

Next, the suitability of U937 cells was assessed by testing the activity of Hla from three different sources (Figure 1D). In‐house produced recombinant Hla (HZI) demonstrated comparable activity (EC_50_ = 5 nM) to Hla from IBT (EC_50_ = 7 nM), while Hla from Sigma exhibited slightly reduced activity (EC_50_ = 15 nM). The latter can be explained by a lower protein content of 60% given for the batch. The assay's validation was further substantiated by using two anti‐Hla antibodies. A concentration‐dependent reversal of Ca^2+^ influx was observed, with higher activity of an anti‐Hla monoclonal antibody in comparison to an anti‐Hla polyclonal antibody (see Figure 1E). The impact of cell number and incubation time on assay efficiency was also investigated and optimized. Specifically, incubation for 4 h in tissue culture treated plates resulted in a high fluorescent signal (Figure 1F) as well as S/B ratios of 5.9 and 6.2 for of 1 × 10^4^ and 2 × 10^4^ cells/well respectively (see Figure 1G). Consequently, 1.2 × 10^4^ cells/well were used in a 1536‐multiwell format to screen > 182,000 compounds with a Z’ factor of 0.79. The compounds were screened at a concentration of 11 µM, and the cutoff for determining a hit or an active compound was defined as 50% reduction in Ca^2+^ influx compared to the DMSO control.

### 2.2 Identification and Synthesis of Thiadiazoles

**SCHEME 1:**
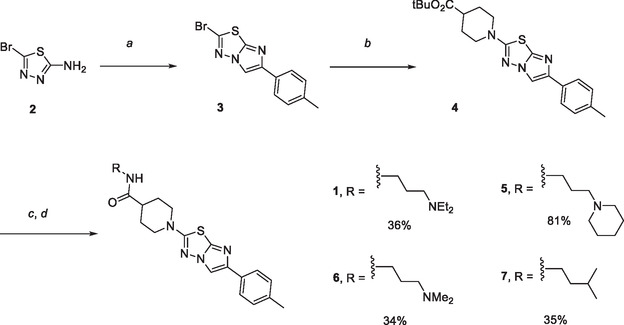
Synthesis of the thiadiazole **1** and its analogs. Reagents and conditions. (a) 2‐bromo‐1‐(*p*‐tolyl)ethan‐1‐one, EtOH, reflux, 16 h, 50%; (b) *tert*‐butyl piperidine‐4‐carboxylate hydrochloride, Et_3_N, EtOH, reflux, 16 h, 36%; (c) TFA, CH_2_Cl_2_, r.t., 3 h; (d) RNH_2_, HATU, DIPEA, THF, DMF, r.t., 18 h.

Among more than 182,000 compounds that were tested in the primary screen, compounds with a 2‐(piperidin‐1‐yl)‐6‐(*p*‐tolyl)imidazo[2,1‐*b*][1,3,4]thiadiazole scaffold—abbreviated as thiadiazoles herein—were identified as active. The compounds are accessible through a short, concise synthesis comprising 4 steps, as exemplified for **1** and its closest analogs (Scheme 1). The heterocyclic core of the inhibitors was assembled by the reaction of 5‐bromo‐1,3,4‐thiadiazol‐2‐amine (**2**) and substituted 2‐bromoacetophenone. The substitution of the bromine substituent of **3** with *tert*‐butyl piperidine‐4‐carboxylate led to the intermediate **4**. The TFA‐mediated cleavage of the *tert*‐butyl protecting group, followed by a HATU coupling with different amines, resulted in the inhibitors **1**, **5**, **6**, and **7**.

**FIGURE 2:**
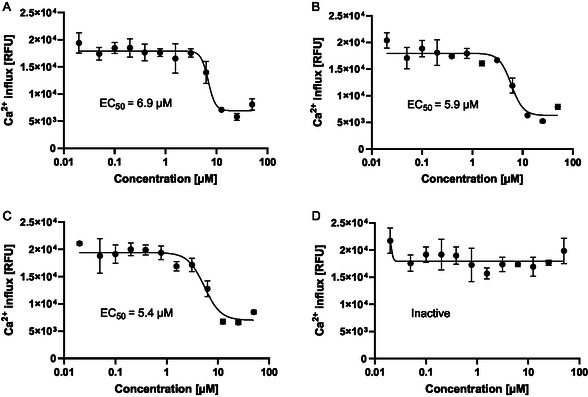
Thiadiazoles prevent *α*‐hemoysin mediated Ca^2+^ influx in U937 monocytes. U937 cells were loaded with Fluo‐4 dye and treated with the indicated compound concentration. Hla (0.5 µg/mL) was added and Ca^2+^ influx was determined after 4 h. EC_50_ of selected analogs **1** (A), **6** (B), and **5** (C) as well as the inactive analog **7** lacking the basic nitrogen atom at the side chain (D) are shown. Data points indicate means ± SD, *n* = 3.

We investigated a series of 18 analogs that differed in the substitution pattern at the piperidine ring (Table 1). A basic nitrogen atom at the side chain extending from the piperidine ring was important for activity, because analogs devoid of it such as **7**, **8**, **9**, **10**, or **11** were inactive (Figure 2). The fact that **5** containing a 4‐substituted piperidine showed higher activity (EC_50_ = 5.4 µM) than its isomer **12** with a 3‐substituted piperidine (EC_50_ = 15 µM) illustrated the relevance of the substitution position of the piperidine moiety. The length of the linker between the amide moiety and the basic nitrogen also influenced activity: Compounds containing a linker of three methylene groups such as **1**, **5**, and **6** (EC_50_ = 6.9, 5.9, and 5.4 µM, respectively) were more active than **13** (inactive), **14** (EC_50_ = 15 µM), and **15** (inactive), matched molecular pairs with two methylene groups. A cyclic linker as in **16** showed moderate potency (EC_50_ = 11 µM). Finally, we assessed substituent effects at the basic nitrogen. Compounds **1**, **5**, and **6** containing Et_2_N‐ and Me_2_N‐ and (CH_2_)_5_N‐ groups show comparable activities (EC_50_'s = 6.9, 5.9, and 5.4 µM, respectively). However, enlarging the piperidine ring to an azepane as in **17** or replacing it by morpholine as in **18** led to a pronounced drop of activity. The unsubstituted analog **19** and the 4‐substituted analogs **20** and **21** with *N*‐methyl‐piperazine or morpholine rests were all moderately active (EC_50_'s = 24, 17, and 21 µM, respectively).

**TABLE 1:** Structures and inhibitory activities of thiadiazoles in an Hla‐induced Ca^2+^ influx assay.

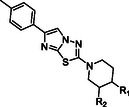			
Compound	R_1_	R_2_	EC_50_, µM
**1**	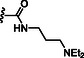	H	6.9
**5**	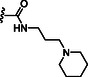	H	5.4
**6**	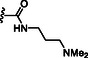	H	5.9
**7**	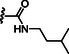	H	Inactive
**8**	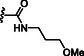	H	>35
**9**	H	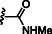	>35
**10**	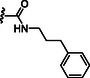	H	>35
**11**	H	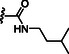	>35
**12**	H	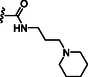	15
**13**	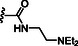	H	>35
**14**	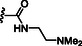	H	15
**15**	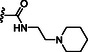	H	>35
**16**	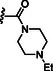	H	11
**17**	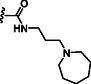	H	25
**18**	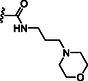	H	>35
**19**	H	H	24
**20**	H	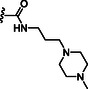	17
**21**	H	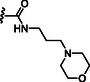	21

In summary, we found that thiadiazoles inhibited Hla in a cellular assay with potencies in the single‐digit µM range. The compounds are readily accessible, and a basic nitrogen, distant from the thiadiazoles core, was essential for activity.

### 2.3 Stability and Plasma Protein Binding

In order to assess the potential of thiadiazoles to become drug leads, we determined the chemical stability and also the absorption, distribution, metabolism, and elimination (ADME) properties of **1** by a combination of in vitro and in silico [21, 22] studies (Supplementary Table 1). In the presence of mouse or human plasma, the majority of **1** (96.1% or 96.4%, respectively) was found bound to plasma proteins. Chemical stability tests showed that **1** was fully stable at acidic and neutral pH values of 1 and 7.4, whereas it showed limited lability at a basic pH of 9, with 83% of compound remaining intact after 24 h. The presence of human or mouse plasma did not impair the stability either. When **1** was exposed to human our mouse liver microsomes, very low clearances of 1 and 7.3 µL/min/mg were observed. The compounds were also insensitive toward phase 2 metabolism, because stabilities of 93.4% and 100% were observed following an incubation with mouse or human liver microsomes at 37°C for 1 h. In conclusion, **1** was highly stable toward chemical or enzymatic degradation in relevant matrices and exhibited high plasma protein binding.

### 2.4 Structure of a Complex Between α‐Hemolysin and 1

**FIGURE 3:**
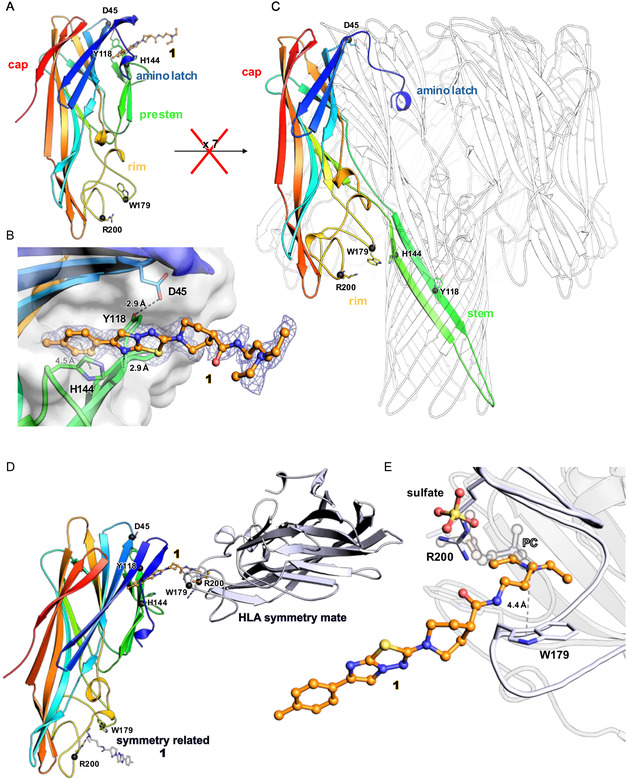
Crystal structure of monomeric Hla in complex with **1**. (A) Hla monomer colored from N‐ to C‐terminus (blue to red) bound to **1** (orange). Residues mechanistically important or involved in binding of **1** are shown as sticks and their C_α_‐carbons are indicated by black spheres. The *N*‐terminal amino latch keeps the prestem *β*‐strand in retracted position. (B) Close‐up view of **1** binding. The blue mesh represents the electron density of an omit map for the compound, contoured at *σ* = 1. The compound forms a single hydrogen bond (dashed black line) to the main chain amide of tyrosine 118 of the prestem, which interacts with aspartate 45 (dashed black line). A *π*/*π* interaction is present to Histidine 144 in the prestem (dahed gray line). **1** supposedly prevents extension of the amino latch and thus assembly of the heptamer. (C) Heptameric Hla pore (PDB: 7AHL [23]). The extended stems form the membrane‐penetrating *β*‐barrel. (D) **1** mediates a crystal contact to another Hla monomer (blue‐gray). (E) Close‐up view of the interaction of **1** with the symmetry related Hla molecule. A potential *π*/cation interaction is shown as dashed gray line. Superimposed (light gray with outline) is a structure of the inactive H35A variant of Hla in complex with a phosphatidylcholine (PC) derivative (PDB: 6u3t [24]). The position of the phosphate in this compound is occupied by a sulfate in the **1** complex structure. Coordinates and structure factors have been deposited in the Protein Data Bank under the identifier 9shr.

To gain more detailed insight into the mode of action of **1**, we determined the crystal structure of an *α*‐hemolysin/inhibitor complex (Figure 3). For this, recombinant wild‐type protein was crystallized in the presence of a 3.5‐fold excess of ligand, resulting in crystals belonging to spacegroup P6_2_22 and diffracting to 2.1 Å resolution ( Table 2). To our surprise, the asymmetric unit of these crystals contained only one monomer of *α*‐hemolysin in a non‐heptameric form. Instead, the structure of the protein is highly similar to the H35A mutant of *α*‐hemolysin (PDB ID 4YHD) [25], a variant known to have significantly impaired oligomerization capabilities and hence hemolysis activity [26]. A hallmark of this monomeric structure is the finding that residues 10–15 of the *N*‐terminal amino latch (residues 1–16) establish a *β*‐sheet‐like interaction with the *N*‐terminal section of the *β*‐strand consisting of residues 18–29, anchoring the latch at the cap domain of *α*‐hemolysin, which otherwise protrudes away in the heptameric pore. Together with the prestem structure, this creates a mainly hydrophobic pocket that accommodates the (*p*‐tolyl)imidazo‐thiadiazole head group of the inhibitor. Here, the inhibitor engages in only one polar contact with the toxin, namely between the basic nitrogen of its imidazole moiety and the backbone amide of Y118. Interestingly, this amino acid has previously been identified as a key residue to stabilize the monomeric form of *α*‐hemolysin by engaging into a hydrogen bond with the side chain of D45 [25]. Indeed, this interaction is preserved in the structure of the inhibitor complex, suggesting that the inhibitor may strengthen the D45/Y118 hydrogen bond. *π*/*π* stacking interactions of the thiadiazole with H144 may further contribute to the binding of **1**. Together, this suggests that the inhibitor blocks the release of the amino latch, thereby impairing pore formation.

The basic side chain of the inhibitor projects points away from the protein, and it is essential for bioactivity. In the crystal structure, it contacts the rim domain of a neighboring molecule, where the tertiary amine binds to crevice on the surface without establishing direct interactions except for a potential cation‐*π*‐bond to W179. Interestingly, this pocket is not only found in Hla, but also conserved in the F‐component of binary pore‐forming toxins of *S. aureus* and has been implicated in the binding of phospholipids [23]. Comparisons to structures of these toxins [27] in complex with phosphatidylcholine (PC) derivatives (H35A‐Hla: PDB ID 6u3t; LukD: 6u2s; Panton‐Valentine leukocydin LukF: 6u3f [24]; *γ*‐hemolysin LukF: 3lkf [28]) show that the tertiary amine of **1** takes a similar position as the quaternary amino group of PC, whereas a bound sulfate ion in the structure presented here mimics the phosphate moiety of PC, interacting with side chain of the conserved R200. Intriguingly, it has recently been shown that PC derivatives protect cells against pore‐forming *S. aureus* toxins, potentially by preventing their binding and/or interfering with pore assembly at the host cell membrane [24]. It is thus conceivable that **1** may act via two mechanisms, namely by securing the *N*‐terminal amino latch and preventing structural rearrangements of the prestem sequence required for pore assembly, and by inhibiting membrane attachment through blocking a phospholipid binding site [24]. The latter mechanism would also explain why the basic Eastern residue was essential for the cellular activity of the thiadiazoles.

## 3 Conclusion

In order to improve the treatment of infections with harmful bacterial pathogens such as *S. aureus*, alternative treatment concepts that go beyond antibiotics are needed. Among those, blocking Hla, the key virulence factor of *S. aureus*, represents an attractive approach that is supported by ample preclinical and even clinical data. This study discloses thiadiazoles as a new class of Hla inhibitors that directly interacted with the toxin. This mode of action is shared with the QDS inhibitors that were recently reported by us. The co‐crystal structure demonstrated a binding mode that is different from that of the QDS series, and it suggests an original dual mechanism that prevents stem loop unfolding as well as membrane attachment. While an extensive QDS optimization program with 900 analogs led to analogs with low nM potency, the thiadiazoles exhibited weaker potency in the low µM range. But yet, the 18 analogs reported here do not address all structural elements. For example, the roles of the arene ring and the central imidazo[2,1‐b][1,3,4]thiadiazole need further investigation in a future, structure‐based hit optimization program to leverage the potential of the series.

## 4 Experimental Section

### 4.1 Materials

**Table:** 

Biological materials	Source	Identifier
Recombinant proteins and antibodies		
Alpha‐hemolysin	IBT Bioservices	Cat# 1401‐002
Alpha‐hemolysin	Sigma–Aldrich	Cat# H9395
Alpha‐hemolysin	This study / HZI	N/A
Mouse anti‐Hla mAb	IBT Bioservices	Cat# 0210‐001
Rabbit anti‐Hla pAb	Sigma–Aldrich	Cat# S7531
Critical commercial assays and kits		
Fluo‐4 NW Calcium assay kit	Life Technologies	Cat# F36206
CyQUANT LDH Cytotoxicity assay	ThermoFisher Scientific	Cat# C20300
Experimental models: cells		
U937 cells	DSMZ	ACC5
A549 cells	DSMZ	ACC107
L929	DSMZ	ACC2
KB3.1	DSMZ	ACC158
RAW264.7	ATCC	TIB‐71
Software and algorithms		
GraphPadPrism 9.5.1	GraphPad	https://www.graphpad.com/
Biorender	Biorender	https://www.biorender.com/
Devices		
Synergy microplate reader	Biotek (Agilent)	N/A
M200 PRO microplate reader	Tecan	N/A
Microplate reader	EnVision, Perkin	N/A

The compounds **1**, **8**–**18**, **20**, and **21** were commercially available and were purchased from ChemDiv.

### 4.2 Chemical Synthesis

#### 4.2.1 Starting Materials

Starting materials were purchased from commercial suppliers (Sigma–Aldrich, TCI, BLDpharm, abcr, Carbolution, Thermo Scientific) and used without further purification.

#### 4.2.2 Accurate Mass Method

High‐resolution masses were obtained using a Maxis II TM HD mass spectrometer (Bruker Daltonics, Bremen, Germany).

#### 4.2.3 Flash Column Chromatography

Purification on reverse phase was done with a Pure C‐850 FlashPrep system (Büchi) using FlashPure EcoFlex C18 cartridges (Büchi). A gradient of water and acetonitrile was used as an eluent. Dryloads were prepared with silica gel C18, 0.035–0.07, 400−220 mesh (Carl Roth).

Normal phase purification was carried out with a Pure C‐810 Flash system (Büchi) using FlashPure cartridges (Büchi). A gradient of cyclohexane and ethyl acetate or dichloromethane and methanol was used as an eluent. Dryloads were prepared with silica gel, 60 Å, 230–400 mesh, 40–63 µm (Merck).

#### 4.2.4 High‐Performance Liquid Chromatography (HPLC)

HPLC was carried out with a Dionex UltiMate 3000 system (Thermo Scientific) using a Luna 5 µm C18(2) 100 Å, LC column 250 × 21.2 mm, AXIA packed (phenomenex). As an eluent, water and acetonitrile were used without or with 0.1% formic acid.

#### 4.2.5 NMR Methods

Proton (^1^H) and carbon (^13^C) nuclear magnetic resonance (NMR) spectra were measured with a Bruker Avance II (300 MHz), Bruker Avance III (500 MHz), Bruker Avance III (600 MHz), or Bruker Avance III HD (700 MHz) spectrometer with residual protonated solvent (DMSO *δ* 2.49) as standard. The NMR data of the synthesized examples are in agreement with their corresponding structural assignments.

#### 4.2.6 2‐Bromo‐6‐(p‐tolyl)imidazo[2,1‐b][1,3,4]thiadiazole (3)

The compound **3** was synthesized according to Kukaniev et al. [29]. A solution of 5‐bromo‐1,3,4‐thiadiazol‐2‐amine (**2**) (845 mg, 4.7 mmol) and 2‐bromo‐1‐(*p*‐tolyl)ethan‐1‐one (1.0 g, 4.7 mmol) in abs. EtOH (30 mL) was stirred at reflux (bath temperature 100°C) for 16 h. The reaction mixture was poured into water. The precipitate was filtered. The crude product was purified by flash chromatography (cyclohexane : EtOAc then CH_2_Cl_2_ : MeOH). Yield: 690 mg (50%).

^1^H NMR (700 MHz, DMSO‐*d*_6_): *δ* = 8.69 (s, 1 H), 7.75 (d, *J* = 8.1 Hz, 2 H), 7.22 (d, *J* = 8.1 Hz, 2 H), 2.32 (s, 3 H) ppm.

^13^C NMR (175 MHz, DMSO‐*d*_6_): *δ* = 145.0, 144.9, 136.8, 134.8, 130.7, 129.3, 124.6, 110.5, 20.8 ppm.

HRMS (ESI) calcd. for C_11_H_9_BrN_3_S [M+H]^+^: *m*/*z* = 293.9701/295.9680, found: 293.9699/295.9677.

#### 4.2.7 *tert*‐Butyl 1‐(6‐(*p*‐Tolyl)imidazo[2,1‐b][1,3,4]thiadiazol‐2‐yl)piperidine‐4‐carboxylate (4)

The compound **3** (690 mg, 2.3 mmol) was dissolved in EtOH (30 mL). *tert*‐Butyl piperidine‐4‐carboxylate hydrochloride (530 mg, 2.4 mmol) and Et_3_N (0.7 mL, 4.8 mmol) were added and reaction mixture was refluxed for 16 h (bath temperature 100°C). The solvent was removed under reduced pressure and the residue was purified by flash chromatography (CH_2_Cl_2_: MeOH). Yield: 326 mg (36%).

^1^H NMR (500 MHz, DMSO‐*d*_6_): *δ* = 8.24 (s, 1 H), 7.66 (d, *J* = 7.8 Hz, 2 H), 7.16 (d, *J* = 7.8 Hz, 2 H), 3.78–3.70 (m, 2 H), 3.27–3.16 (m, 2 H), 2.60–2.53 (m, 1 H), 2.29 (s, 3 H), 1.97–1.88 (m, 2 H), 1.70–1.56 (m, 2 H), 1.41 (s, 9 H) ppm.

^13^C NMR (125 MHz, DMSO‐*d*_6_): *δ* = 172.9, 164.2, 142.5, 135.6, 131.6, 129.1, 124.1, 109.5, 79.9, 47.5, 27.7, 26.8, 20.8 ppm.

HRMS (ESI) calcd. for C_21_H_27_N_4_O_2_S [M+H]^+^: *m*/*z* = 399.1855, found: 399.1847.

#### 4.2.8 2‐(Piperidin‐1‐yl)‐6‐(p‐tolyl)imidazo[2,1‐b][1,3,4]thiadiazole (19)

The compound **3** (50 mg, 0.17 mmol) was stirred in piperidine in a closed vial at 150°C. The product was used without any further purification. Yield: 31 mg (61%).

^1^H NMR (300 MHz, CDCl_3_): *δ* = 7.62 (s, 1 H), 7.59 (d, *J* = 8.0 Hz, 2 H), 7.12 (d, *J* = 8.0 Hz, 2 H), 3.41–3.32 (m, 4 H), 2.29 (s, 3 H), 1.72–1.54 (m, 6 H) ppm.

MS (ESI) calcd. for C_16_H_19_N_4_S [M+H]: m/*z* = 299, found: 299.

#### 4.2.9 General Procedure 1 (GP1) for the Synthesis of *N*‐Substituted 1‐(6‐(*p*‐Tolyl)imidazo[2,1‐b][1,3,4]thiadiazol‐2‐yl)piperidine‐4‐carboxamides

To a mixture of the compound **4** (50 mg, 0.13 mmol) and CH_2_Cl_2_ (2 mL) was added TFA (1 mL). The reaction mixture was stirred for 3 h at r. t. Volatiles were removed under reduced pressure, and DMF (1 mL) was added to the residue. The solution of the corresponding amine (0.16 mmol) in THF (3 mL) and DIPEA (0.18 mL, 1.0 mmol) was added followed by HATU (76 mg, 0.2 mmol). The reaction mixture was stirred for 18 h at r.t. Aqueous NaOH (1M, 25 mL) was added and the mixture was stirred for 1 h at r.t. The mixture was extracted with EtOAc (3x), filtered, and concentrated under reduced pressure The residue was purified by flash chromatography on reversed phase (C18; MeCN : H_2_O).

#### 4.2.10 *N*‐(3‐(Diethylamino)propyl)‐1‐(6‐(*p*‐tolyl)imidazo[2,1‐b][1,3,4]thiadiazol‐2‐yl)piperidine‐4‐carboxamide (1)

The compound **1** was obtained according to GP1 starting from **4** (50 mg, 0.13 mmol), *N* ^1^,*N* ^1^‐diethylpropane‐1,3‐diamine (21 mg, 0.16 mmol) and HATU (76 mg, 0.2 mmol). Yield: 21 mg (36%).

^1^H NMR (500 MHz, DMSO‐*d*_6_): *δ* = 8.24 (s, 1 H), 7.85 (t, *J* = 5.5 Hz, 1 H), 7.67 (d, *J* = 8.0 Hz, 2 H), 7.17 (d, *J* = 8.0 Hz, 2 H), 3.80 (d, *J* = 13.0 Hz, 2 H), 3.17 (td, *J* = 12.4, 2.7 Hz, 2 H), 3.08–3.02 (m, 2 H), 2.45–2.38 (m, 1 H), 2.41 (q, *J* = 7.1 Hz, 4 H), 2.37–2.32 (m, 2 H), 2.30 (s, 3 H), 1.83–1.76 (m, 2 H), 1.70–1.59 (m, 2 H), 1.53–1.46 (m, 2 H), 0.92 (t, *J* = 7.1 Hz, 6 H) ppm.

^13^C NMR (125 MHz, DMSO‐*d*_6_): *δ* = 173.2, 164.2, 142.5, 140.4, 135.7, 131.6, 129.1, 124.1, 109.5, 49.9, 47.8, 46.3, 41.0, 37.0, 27.4, 26.6, 20.8, 11.7 ppm.

HRMS (ESI) calcd. for C_24_H_35_N_6_OS [M+H]^+^: *m*/*z* = 455.2593, found: 455.2588.

#### 4.2.11 *N*‐(3‐(Piperidin‐1‐yl)propyl)‐1‐(6‐(*p*‐tolyl)imidazo[2,1‐b][1,3,4]thiadiazol‐2‐yl)piperidine‐4‐carboxamide (5)

The compound **5** was obtained according to GP1 starting from **4** (50 mg, 0.13 mmol), 3‐(piperidin‐1‐yl)propan‐1‐amine (23 mg, 0.16 mmol), and HATU (76 mg, 0.2 mmol). Yield: 49 mg (81%).

^1^H NMR (500 MHz, DMSO‐*d*_6_): *δ* = 8.24 (s, 1 H), 7.86 (t, *J* = 5.5 Hz, 1 H), 7.67 (d, *J* = 8.0 Hz, 2 H), 7.17 (d, *J* = 8.0 Hz, 2 H), 3.80 (d, *J* = 13.0 Hz, 2 H), 3.17 (td, *J* = 12.4, 2.7 Hz, 2 H), 3.07–3.01 (m, 2 H), 2.43–2.35 (m, 1 H), 2.32–2.24 (m, 4 H), 2.30 (s, 3 H), 2.21 (t, *J* = 7.2 Hz, 2 H), 1.83–1.76 (m, 2 H), 1.70–1.59 (m, 2 H), 1.56–1.51 (m, 2 H), 1.50–1.44 (m, 4 H), 1.39–1.32 (m, 2 H) ppm.

^13^C NMR (125 MHz, DMSO‐*d*_6_): *δ* = 173.2, 164.2, 142.5, 140.4, 135.6, 131.6, 129.1, 124.1, 109.5, 56.3, 54.1, 47.8, 41.0, 37.1, 27.4, 26.4, 25.6, 24.1, 20.8 ppm.

HRMS (ESI) calcd. for C_25_H_35_N_6_OS [M+H]^+^: *m*/*z* = 467.2593, found: 467.2605.

#### 4.2.12 *N*‐(3‐(Dimethylamino)propyl)‐1‐(6‐(*p*‐tolyl)imidazo[2,1‐b][1,3,4]thiadiazol‐2‐yl)piperidine‐4‐carboxamide (6)

The compound **6** was obtained according to GP1 starting from **4** (50 mg, 0.13 mmol), *N* ^1^,*N* ^1^‐dimethylpropane‐1,3‐diamine (17 mg, 0.16 mmol), and HATU (76 mg, 0.2 mmol). Yield: 19 mg (34%).

^1^H NMR (600 MHz, DMSO‐*d*_6_): *δ* = 8.24 (s, 1 H), 7.87 (t, *J* = 5.5 Hz, 1 H), 7.67 (d, *J* = 8.0 Hz, 2 H), 7.17 (d, *J* = 8.0 Hz, 2 H), 3.81 (d, *J* = 13.0 Hz, 2 H), 3.17 (td, *J* = 12.4, 2.7 Hz, 2 H), 3.08–3.03 (m, 2 H), 2.40 (tt, *J* = 11.4, 3.6 Hz, 1 H), 2.30 (s, 3 H), 2.24 (t, *J* = 7.0 Hz, 2 H), 2.14 (s, 6 H), 1.83–1.77 (m, 2 H), 1.69–1.60 (m, 2 H), 1.56–1.49 (m, 2 H) ppm.

^13^C NMR (150 MHz, DMSO‐*d*_6_): *δ* = 173.3, 164.2, 142.5, 140.4, 135.7, 131.6, 129.1, 124.1, 109.5, 56.6, 47.8, 45.0, 41.0, 36.7, 27.4, 26.9, 20.8 ppm.

HRMS (ESI) calcd. for C_22_H_31_N_6_OS [M+H]^+^: *m*/*z* = 427.2280, found: 427.2280.

#### 4.2.13 *N*‐Isopentyl‐1‐(6‐(*p*‐tolyl)imidazo[2,1‐b][1,3,4]thiadiazol‐2‐yl)piperidine‐4‐carboxamide (7)

The compound **7** was obtained according to GP1 starting from **4** (50 mg, 0.13 mmol), 3‐methylbutan‐1‐amine hydrochloride (20 mg, 0.16 mmol), and HATU (76 mg, 0.2 mmol). Yield: 19 mg (35%).

^1^H NMR (500 MHz, DMSO‐*d*_6_): *δ* = 8.24 (s, 1 H), 7.80 (t, *J* = 5.6 Hz, 1 H), 7.67 (d, *J* = 8.0 Hz, 2 H), 7.17 (d, *J* = 8.0 Hz, 2 H), 3.80 (d, *J* = 13.0 Hz, 2 H), 3.16 (td, *J* = 12.4, 2.7 Hz, 2 H), 3.09–3.02 (m, 2 H), 2.39 (tt, *J* = 11.4, 3.7 Hz, 1 H), 2.30 (s, 3 H), 1.83–1.75 (m, 2 H), 1.70–1.59 (m, 2 H), 1.59–1.51 (m, 1 H), 1.32–1.25 (m, 2 H), 0.86 (d, *J* = 6.6 Hz, 6 H) ppm.

^13^C NMR (125 MHz, DMSO‐*d*_6_): *δ* = 173.2, 164.2, 142.5, 140.4, 135.6, 131.6, 129.1, 124.1, 109.5, 47.8, 41.0, 38.1, 36.6, 27.4, 25.1, 22.4, 20.8 ppm.

HRMS (ESI) calcd. for C_22_H_30_N_5_OS [M+H]^+^: *m*/*z* = 412.2171, found: 412.2163.

### 4.3 Computational Predictions

Predictions of properties were made using the Schrödinger platform “LiveDesign” (Schrödinger Release 2023‐2: LiveDesign, Schrödinger, LLC, New York, NY, 2023), integrated with the ChemAxon Computational Calculator, Product version 16.7.4.0, Chemaxon (https://www.chemaxon.com) [21, 22].

### 4.4 Assay Development

Calcium influx was determined by employing the Fluo‐4 NW calcium assay kit (Life Technologies) as per the manufacturer's instructions. Cells were incubated with the Fluo‐4 indicator solution in assay buffer supplemented with probenecid and incubated at 37 °C for 30 min followed by incubation at room temperature for 30 min. Compounds were added to wells at given concentrations followed by the addition of Hla. Calcium influx was determined by measuring the fluorescence at an excitation wavelength of 485 nm and emission wavelength of 535 nm. Initially, different cell lines, A549 (DSMZ #ACC107), L929 (DSMZ #ACC2), KB3.1 (DSMZ #ACC158), RAW 264.7 (ATCC #TIB‐71), and U937 (DSMZ #ACC5), were tested in the assay for Hla activity, following which U937 cells were selected for further assay optimization. The assay was performed in 96‐well half area plates with 6 × 10^4^ cells/well and a final assay volume of 50 µL. Further optimization steps involved transfer to a 384‐well plate wherein different cell numbers and incubation times were tested.

Thereafter, the assay was adapted to a 1536‐well format with 1.2 × 10^4^ cells/well and a final volume of 9 µL. Compounds were screened at a concentration of 11 µM against 13 nM Hla with a signal to background (S/B) ratio of 3.75, signal to noise (S/N) ratio of 30.2, and a *Z*’ factor > 0.5. The high‐throughput screening consisted of a combined compound library from HZI (22,763 compounds), LDC/COMAS (159,359 compounds).

### 4.5 Production of Recombinant Hla

The plasmid containing the gene encoding for *S. aureus* *α*‐hemolysin without signal peptide and with a C‐terminal Strep‐tag II was a gift from Prof. S. Engelmann (HZI) and was based on the vector pPR‐IBA1 (IBA). The protein was expressed in *E. coli* BL21 (DE3) in ZYM‐5052 auto‐inducing medium [30] at 20°C for 20–24 h. The cell pellet was resuspended in a buffer containing 20 mM Tris/HCl pH 8.5, 300 mM NaCl, one tablet of complete EDTA‐free protease inhibitor cocktail (Roche), and lysed by sonication. The protein was isolated from the supernatant after centrifugation for 1 h at 100,000×g using a self‐packed 10 mL column with Strep‐Tactin Superflow High‐Capacity resin (IBA) and eluted from the column with a single step of 5 mM D‐desthiobiotin. Gel filtration was carried out using a HiLoad 16/600 Superdex 75 pg column (GE Healthcare/Cytiva) in 20 mM Tris/HCl pH 8.5, 300 mM NaCl. All chromatography steps were carried out using an Äkta Purifier system (GE Healthcare). The peak fractions of the size exclusion were analyzed by SDS‐PAGE, concentrated and flash‐frozen in liquid nitrogen, and stored at −80°C until crystallization screening.

### 4.6 Crystallization

Crystallization trials were set up at room temperature with a HoneyBee 961 crystallization robot (Digilab Genomic Solutions) in Intelli 96‐3 plates (Art Robbins Instruments) with 200 nL protein solution and 200 nL reservoir solution. Crystals were obtained at room temperature in co‐crystallization setups with concentrations of 2.5 mg/mL protein, 250 µM **1**, and 5% (v/v) DMSO. Optimized crystals of sufficient quality for data collection were obtained in 2 M ammonium sulfate, 0.1 M Lithium sulfate, and 18.3% (v/v) glycerol. Crystals were flash‐cooled after harvesting and stored in liquid nitrogen until measuring.

### 4.7 Data Collection and Processing

Data collection was performed at beamline P11 at the Petra III storage ring (Deutsches Elektronen‐Synchrotron, Hamburg, Germany) [31]. All datasets were recorded at a temperature of 100 K. Data processing was carried out using the AutoPROC [32] toolbox (Global Phasing) executing XDS [33], Pointless [34], and Aimless [35].

### 4.8 Structure Determination, Refinement, and Model Building

The structure of the *α*‐hemolysin ligand‐complex was determined by molecular replacement using the available structure of the monomeric H35A variant from the PDB (4YHD [25]) as a search‐model for Phaser [36] from the Phenix suite [37]. The structural models were built using Coot [38] and crystallographic refinement was performed with Phenix.refine [39] including the addition of hydrogens in riding positions and TLS‐refinement. 5% of random reflections were flagged for the calculation of R_free_. The model of monomeric wild‐type *α*‐hemolysin in complex with **1** was at 2.1 Å resolution refined to R/R_free_ of 24/28% in space group P6_2_22. Data collection and refinement statistics are summarized in Table S1. Figures of crystal structures were prepared using the PyMOL Molecular Graphics System version 3.1.6 (Schrödinger, LLC).

Coordinates and structure factors have been deposited in the Protein Data Bank under the identifier 9shr.

## Supporting Information

Additional supporting information can be found online in the Supporting Information section. **Supporting Table S1:** Measured and calculated ADME and physicochemical properties of compound **1**. **Supporting** **Table S2:** X‐ray data collection and refinement statistics.

## Funding

The work was supported by the Helmholtz validation fund (HVFLDC‐003) and the pre‐4D fund of the HZI. We also acknowledge infrastructure support from EU‐OPENSCREEN Impulse initiative (Grant agreement number: 101132028) and the COST Action EURESTOP, CA21145, supported by COST (European Cooperation in Science and Technology).

## Conflicts of Interest

The authors declare no conflicts of interest.

## Supporting information

Supplementary Material

## Data Availability

The data that support the findings of this study are openly available in [Protein Data Bank] at [https://www.rcsb.org], reference number [0].
