# Supplementary material for: An Imidazo[2,1‐b][1,3,4]thiadiazole Derivative Inhibits the Virulence Factor α‐Hemolysin by Blocking the Pullout of Its Stem Domain
Source: ChemMedChem. 2026 Feb 23;21(4):e202501098. doi: 10.1002/cmdc.202501098 (PMC12926788; doi:10.1002/cmdc.202501098)

Supporting Information

An imidazo[2,1-b][1,3,4]thiadiazole derivative inhibits the virulence factor α-hemolysin by blocking the pullout of its stem domain

Peer Lukat, Vadim S. Korotkov, Raffaella Di Lucrezia, Aditya Shekhar, Carsten Degenhart, Randi Diestel, Ursula Bilitewski, Klaus Dinkel, Wulf Blankenfeldt, Mark Brönstrup

Supplementary Table 1. Measured and calculated ADME and physicochemical properties of compound **1**.

Parameter	Value	Method
Plasma Protein Binding mouse (%Bound@5.0µM) [%]	96.1	experimental
Plasma Protein Binding human (%Bound@5.0µM) [%]	96.4	experimental
Chemical Stability (%Remain pH 1@2.0µM) [%]	125	experimental
Chemical Stability (%Remain pH 7.4@2.0µM) [%]	114	experimental
Chemical Stability (%Remain pH 9@2.0µM) [%]	83.1	experimental
Plasma Stability mouse (%Remain@5.0µM) [%]	101.7	experimental
Plasma Stability human (%Remain@5.0µM) [%]	102.5	experimental
Microsomal Stability Phase I mouse (CLint) [µl/min/mg]	7.3	experimental
Microsomal Stability Phase I human (CLint) [µl/min/mg]	1	experimental
Microsomal Stability Phase II mouse (%Remain@5.0µM) [%]	93.4	experimental
Microsomal Stability Phase II human (%Remain@5.0µM) [%]	131.1	experimental
cLogP (cLogP)	3.83	computational
CanvasMolDescriptors (PSA)	94.5	computational
CanvasMolDescriptors (HBD(Lipinsky))	1	computational
CanvasMolDescriptors (HBA(canvas))	3	computational
Qikprop (Polar surface area)	68.19	computational
ChemAxon Props (logP)	4.4	computational
ChemAxon Props (logD[pH=7.4])	2	computational
ChemAxon Props (pKa1)	15.54	computational
ChemAxon Props (pKb1)	9.84	computational
ChemAxon Props (pKb2)	3.3	computational
Qikprop (Solvent accessible surface area)	843.12	computational

Supplementary Table 2. X-ray data collection and refinement statistics.

**Structure**	α-Hemolysin (monomer) + **1**
PDB-ID:	9SHR
**Data collection**	
Beamline	DESY P11
Wavelength (Å)	1.03
Space group	$P6_222$
Cell dimensions	
a, *b*, *c* (Å)	95.76, 95.76, 157.65
α, β, γ (°)	90, 90, 120
Resolution (Å)[a]	82.94 – 2.08 (2.12 – 2.08)
R_{merge} (%)[a]	50.1 (163.0)
R_{pim} (%)[a]	7.5 (34.9)
$I/\sigma I$[a]	14.6 (2.7)
Completeness (%)[a]	100 (100)
Redundancy[a]	48.6 (22.6)
$CC_{1/2}$ (%)[a]	98.3 (40.2)
**Refinement**	
Resolution (Å)	2.08
No. reflections[a]	26287 (2541)
R_{work}/R_{free} (%)	23.94/27.93
No. atoms	2904
Protein	2604
Ligand/others	33/64
Water	203
B-factors (Å^2)	16.32
Protein	15.62
Ligand/others	23.72/28.86
Water	20.07
R.m.s deviations	
Bond lengths (Å)	0.003
Bond angles (º)	0.56
Ramachandran statistics (%)	
Favored	95.79
Allowed	3.86
Outliers	0.35
Clashscore (MolProbity)	1.90
MolProbity score	1.25

[a]Values for the highest resolution shell are shown in parentheses.

NMR Spectra

2-Bromo-6-(p-tolyl)imidazo[2,1-b][1,3,4]thiadiazole (3)

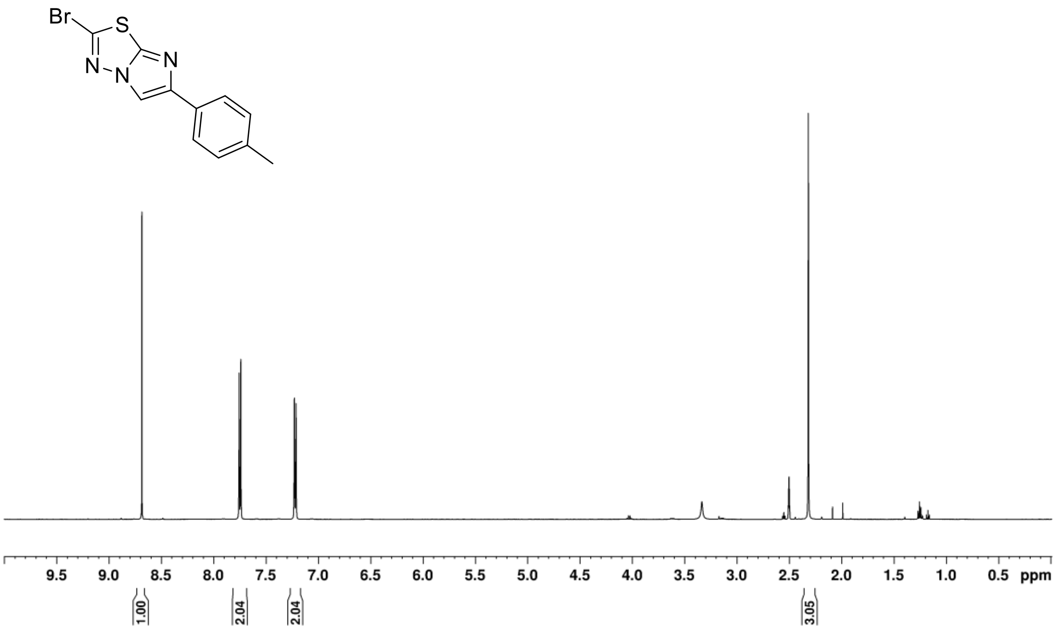

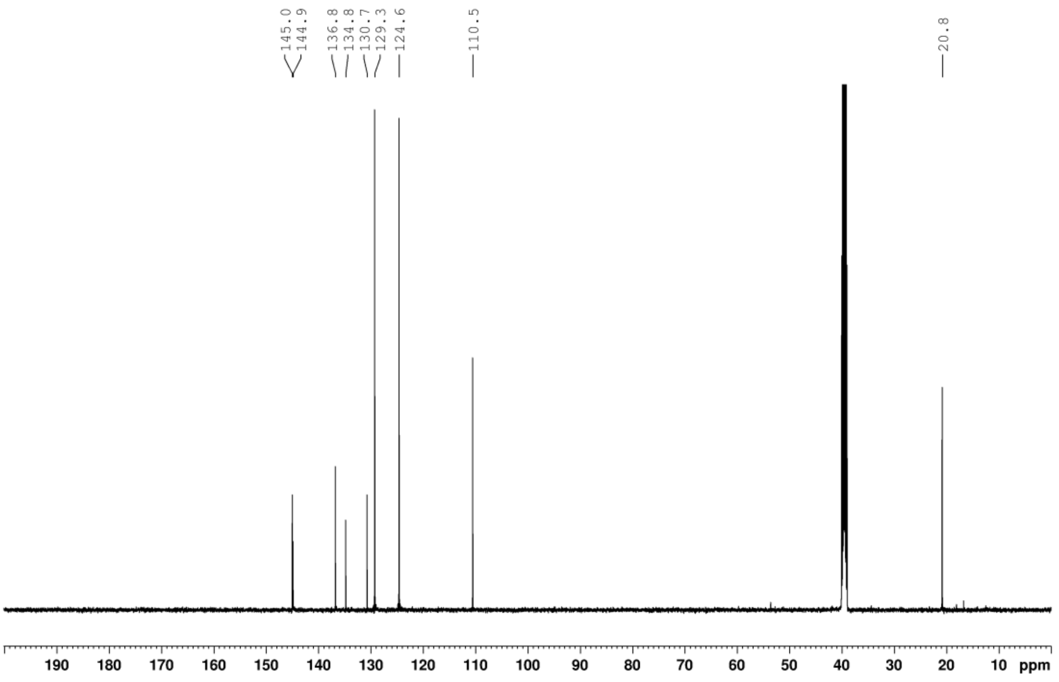

***tert*-Butyl 1-(6-(*p*-Tolyl)imidazo[2,1-b][1,3,4]thiadiazol-2-yl)piperidine-4-carboxylate (4)**

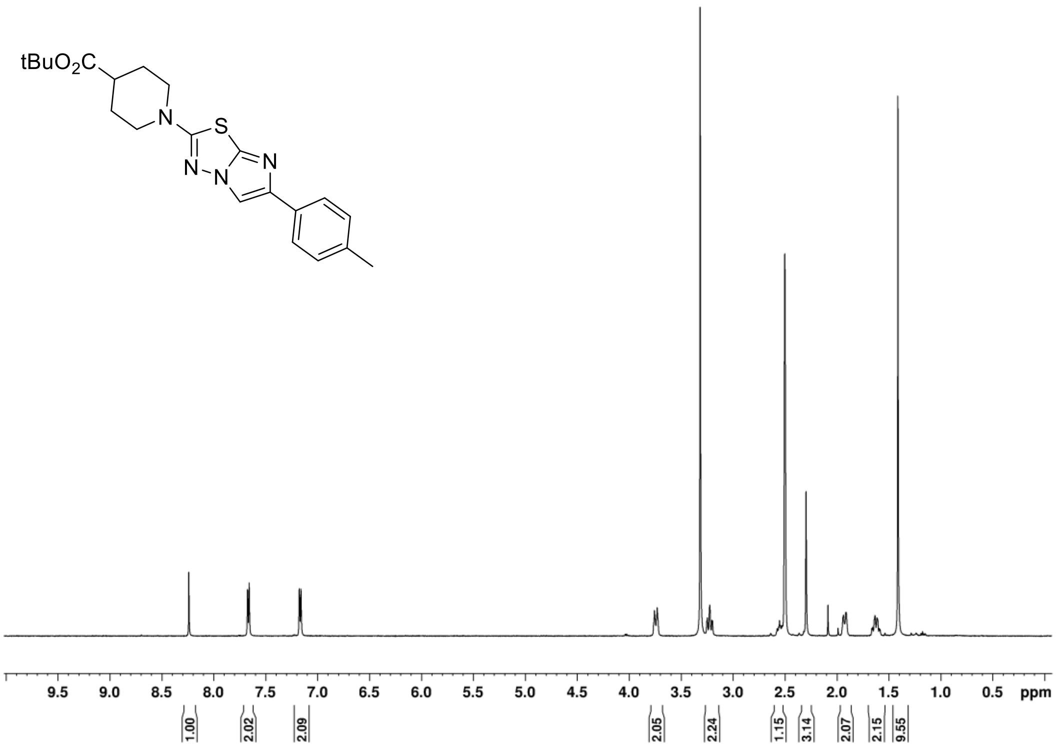

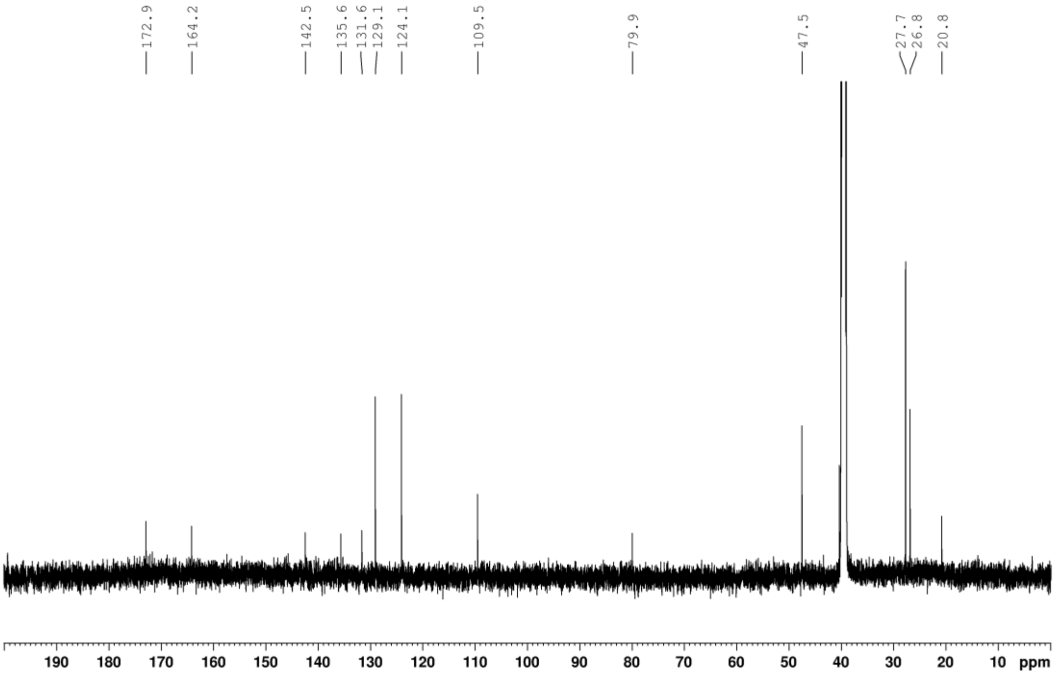

***N*-(3-(Diethylamino)propyl)-1-(6-(p-tolyl)imidazo[2,1-b][1,3,4]thiadiazol-2-yl)piperidine-4-carboxamide (1)**

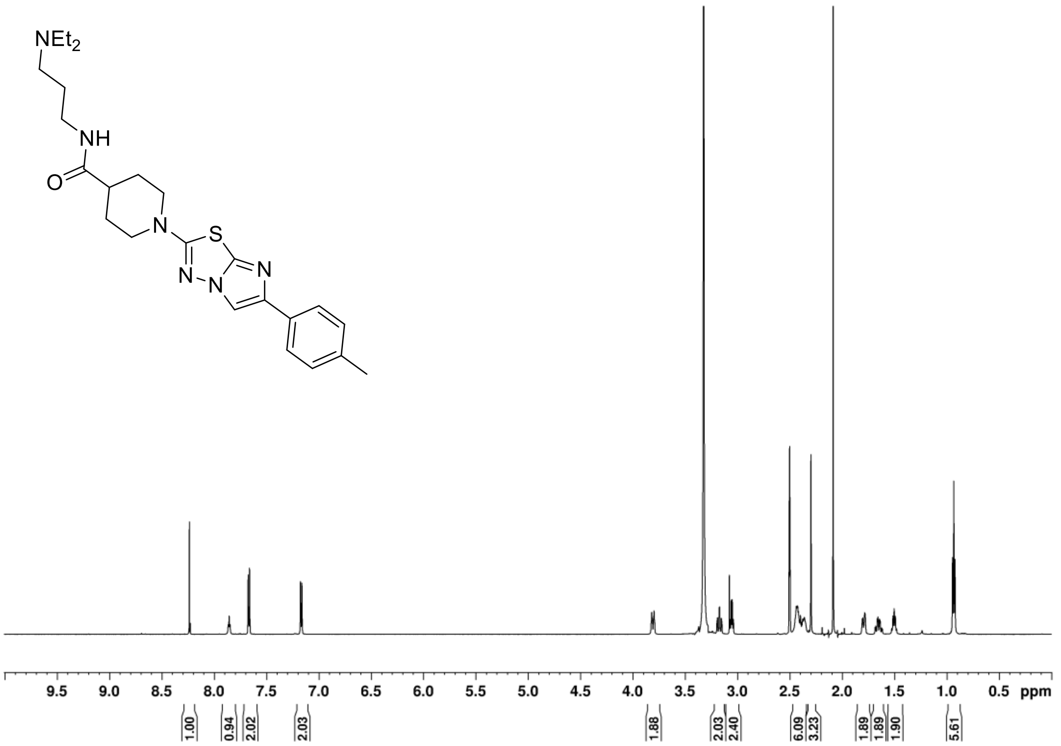

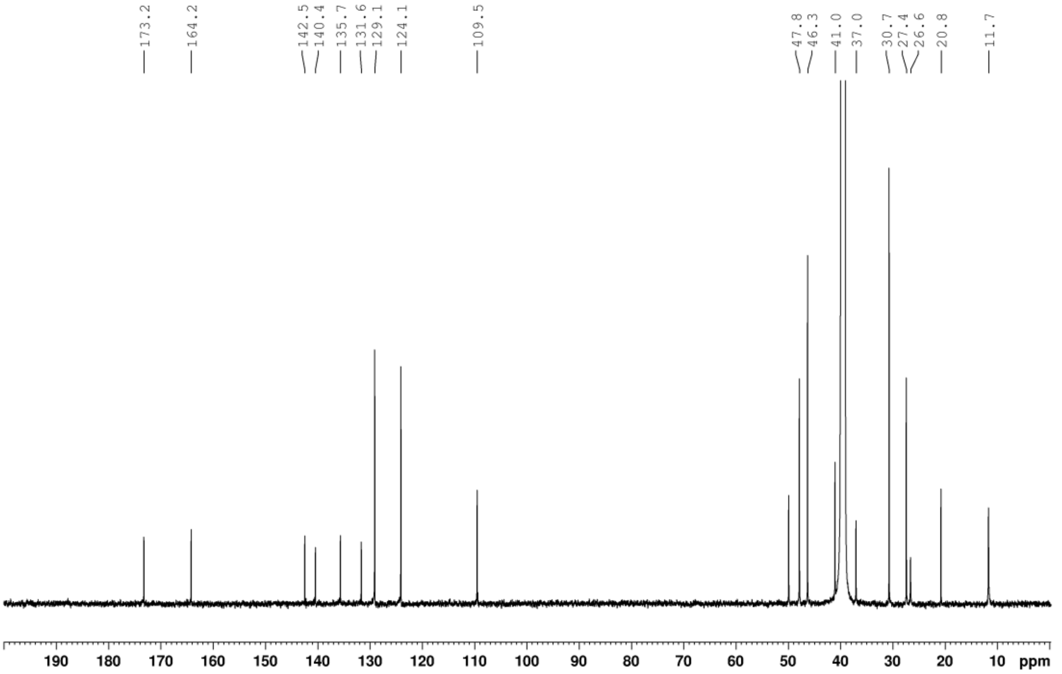

N-(3-(Piperidin-1-yl)propyl)-1-(6-(p-tolyl)imidazo[2,1-b][1,3,4]thiadiazol-2-yl)piperidine-4-carboxamide (5)

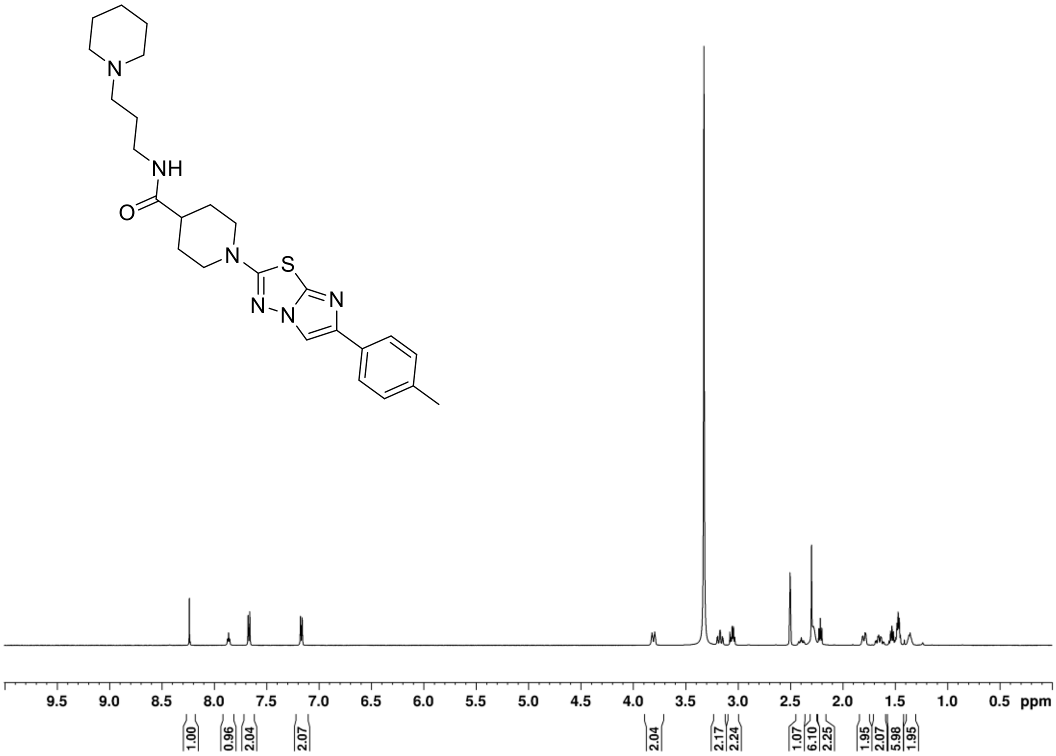

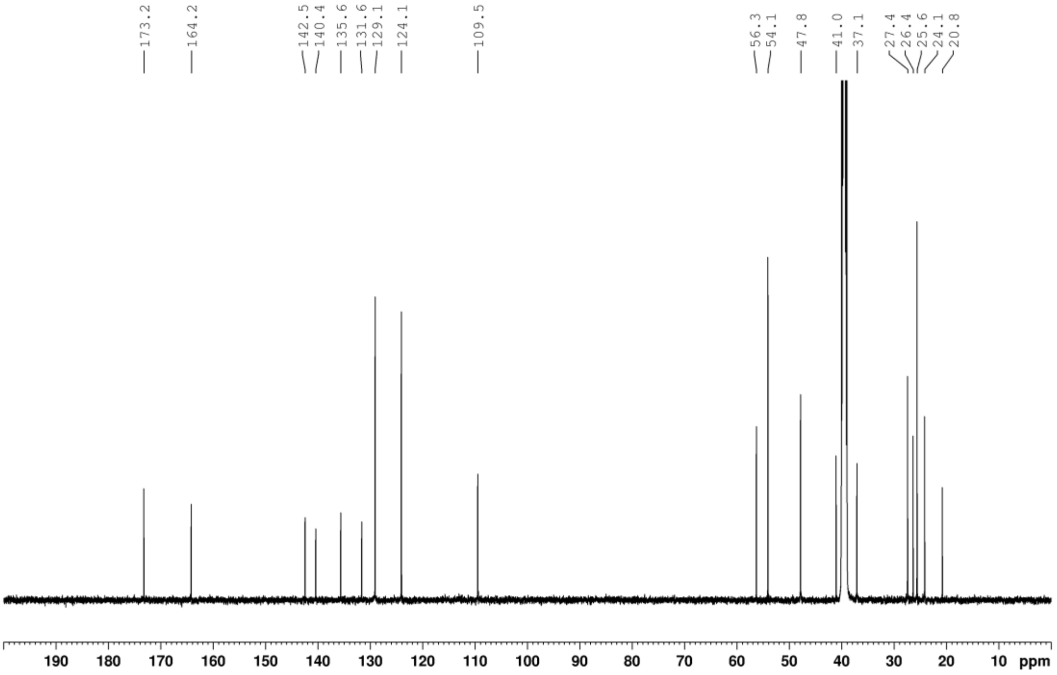

_N_-(3-(Dimethylamino)propyl)-1-(6-(p-tolyl)imidazo[2,1-b][1,3,4]thiadiazol-2-yl)piperidine-4-carboxamide (6)

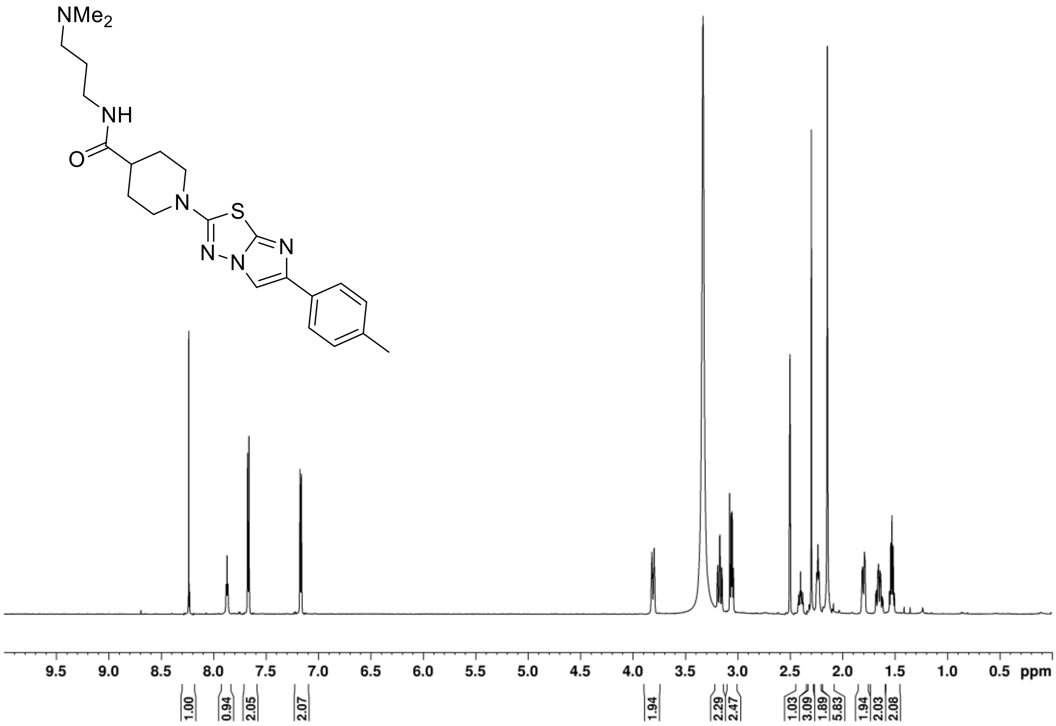

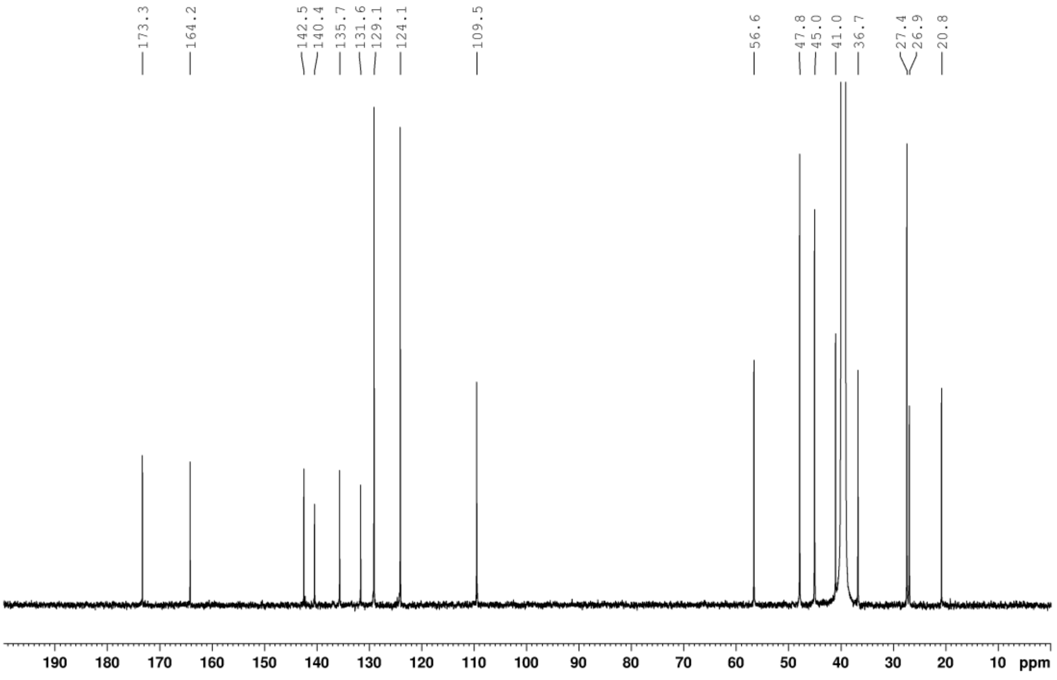

***N*-Isopentyl-1-(6-(p-tolyl)imidazo[2,1-b][1,3,4]thiadiazol-2-yl)piperidine-4-carboxamide (7)**

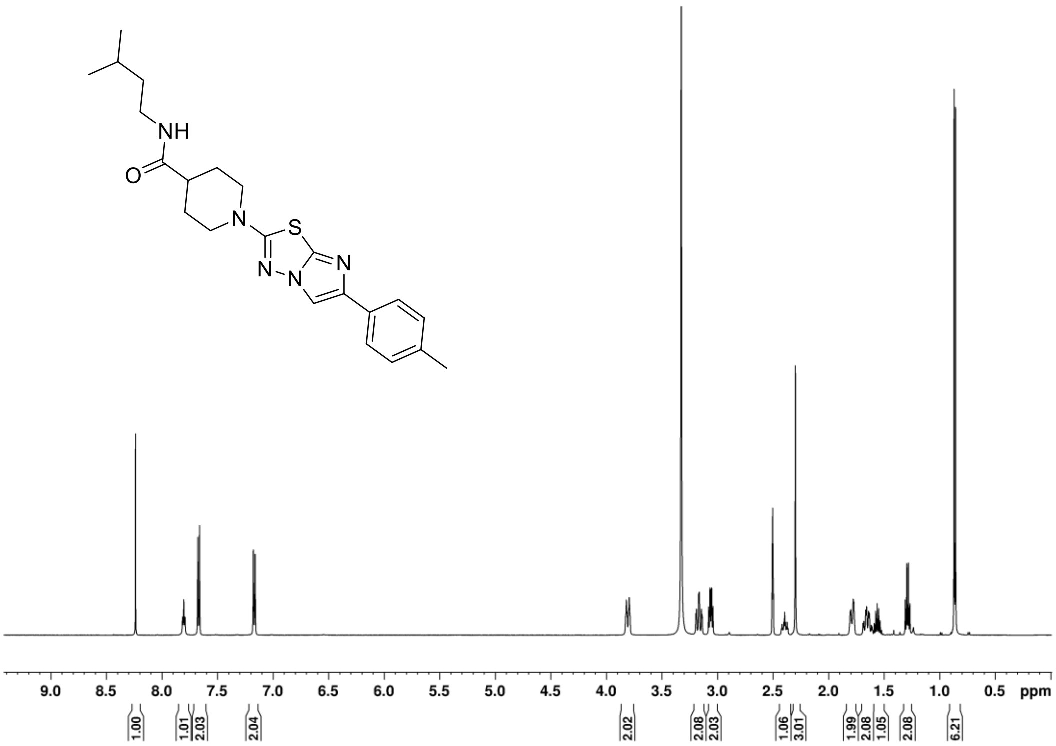

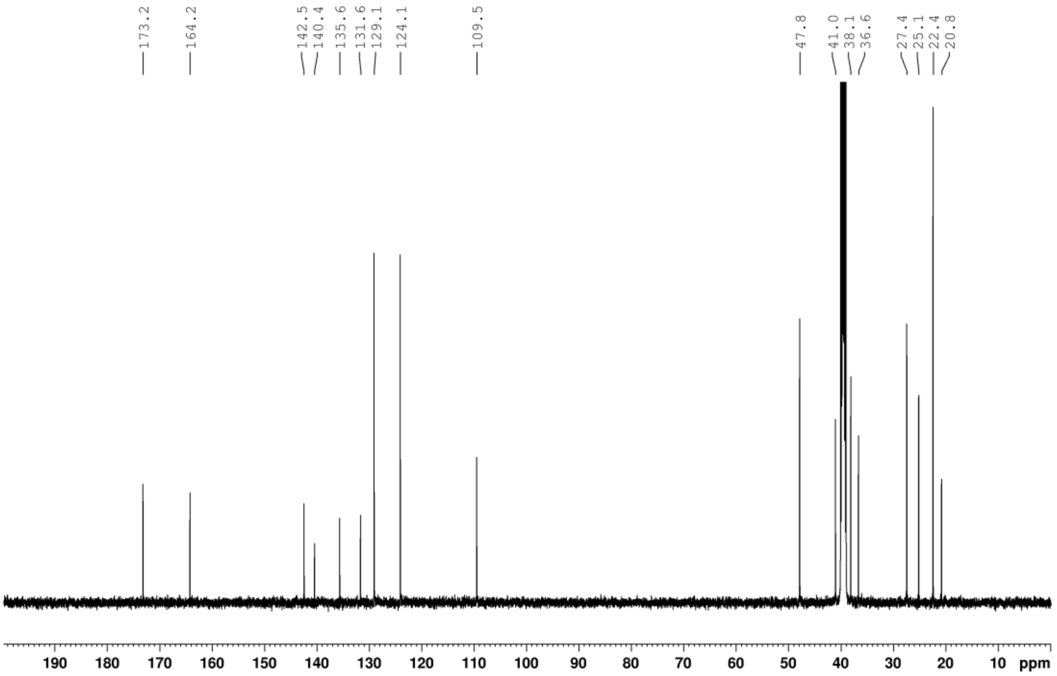

Supplement: Supplementary file 1 — Supplementary Material [file CMDC-21-e202501098-s001.pdf]
